# Publisher Correction: Deciphering photocarrier dynamics for tuneable high-performance perovskite-organic semiconductor heterojunction phototransistors

**DOI:** 10.1038/s41467-020-16563-4

**Published:** 2020-06-08

**Authors:** Yen-Hung Lin, Wentao Huang, Pichaya Pattanasattayavong, Jongchul Lim, Ruipeng Li, Nobuya Sakai, Julianna Panidi, Min Ji Hong, Chun Ma, Nini Wei, Nimer Wehbe, Zhuping Fei, Martin Heeney, John G. Labram, Thomas D. Anthopoulos, Henry J. Snaith

**Affiliations:** 1 Clarendon Laboratory, Department of Physics, University of Oxford, Parks Road, Oxford, OX1 3PU UK; 2 Department of Physics and Centre for Plastic Electronics, Imperial College London, London, SW7 2AZ UK; 3 Department of Materials Science and Engineering, School of Molecular Science and Engineering, Vidyasirimedhi Institute of Science and Technology (VISTEC), Rayong, 21210 Thailand; 4 National Synchrotron Light Source II, Brookhaven National Laboratory, Upton, New York, 11973 USA; 5 School of Electrical Engineering and Computer Science, Oregon State University, Corvallis, OR 97331 USA; 6 Physical Sciences and Engineering Division, King Abdullah University of Science and Technology (KAUST), Thuwal, 23955-6900 Saudi Arabia; 7 King Abdullah University of Science and Technology (KAUST), Core Labs, Thuwal, 23955-6900 Saudi Arabia; 8 Department of Chemistry and Centre for Plastic Electronics, Imperial College London, London, SW7 2AZ UK; 9 Institute of Molecular Plus, Tianjin Key Laboratory of Molecular Optoelectronic Science, Tianjin University, Tianjin, 300072 China

**Keywords:** Electrical and electronic engineering, Electronic properties and materials, Semiconductors, Electronic and spintronic devices

Correction to: *Nature Communications* 10.1038/s41467-019-12481-2, published online 2 October 2019.

In the original version of this article, the unit in the right y-axis of Fig. 4c was mislabelled as 10^12^. The unit should be 10^17^. This has been corrected in the PDF and HTML versions of the Article.

