# Supplementary material for: Deciphering photocarrier dynamics for tuneable high-performance perovskite-organic semiconductor heterojunction phototransistors
Source: Nat Commun. 2019 Oct 2;10:4475. doi: 10.1038/s41467-019-12481-2 (PMC6775060; doi:10.1038/s41467-019-12481-2)
Supplement: Supplementary file 1 — Supplementary Information [file 41467_2019_12481_MOESM1_ESM.pdf]

Supplementary Information

Deciphering photocarrier dynamics for tuneable high-performance perovskite-organic semiconductor heterojunction phototransistors

Yen-Hung Lin[1†]*, Wentao Huang[2†], Pichaya Pattanasattayavong[3], Jongchul Lim[1], Ruipeng Li[4], Nobuya Sakai[1], Julianna Panidi[2], Min Ji Hong[5], Chun Ma[6], Nini Wei[7], Nimer Wehbe[7], Zhuping Fei[8,9], Martin Heeney[8], John G. Labram[5], Thomas D. Anthopoulos[6]*, Henry J. Snaith[1]*

[1]Clarendon Laboratory, Department of Physics, University of Oxford, Parks Road, Oxford OX1 3PU, UK

[2]Department of Physics and Centre for Plastic Electronics, Imperial College London, London SW7 2AZ, UK

[3]Department of Materials Science and Engineering, School of Molecular Science and Engineering, Vidyasirimedhi Institute of Science and Technology (VISTEC), Rayong 21210, Thailand

[4]National Synchrotron Light Source II, Brookhaven National Laboratory, Upton, New York 11973, USA

[5]School of Electrical Engineering and Computer Science, Oregon State University, Corvallis, OR 97331, USA

[6]Physical Sciences and Engineering Division, King Abdullah University of Science and Technology (KAUST), Thuwal 23955-6900, Saudi Arabia

[7]King Abdullah University of Science and Technology (KAUST), Core Labs, Thuwal, 23955-6900, Saudi Arabia

[8]Department of Chemistry and Centre for Plastic Electronics, Imperial College London, London SW7 2AZ, UK

[9]Institute of Molecular Plus, Tianjin Key Laboratory of Molecular Optoelectronic Science, Tianjin University, Tianjin 300072, China

*Correspondence: henry.snaith@physics.ox.ac.uk; thomas.anthopoulos@kaust.edu.sa; yen-hung.lin@physics.ox.ac.uk

Supplementary Figures

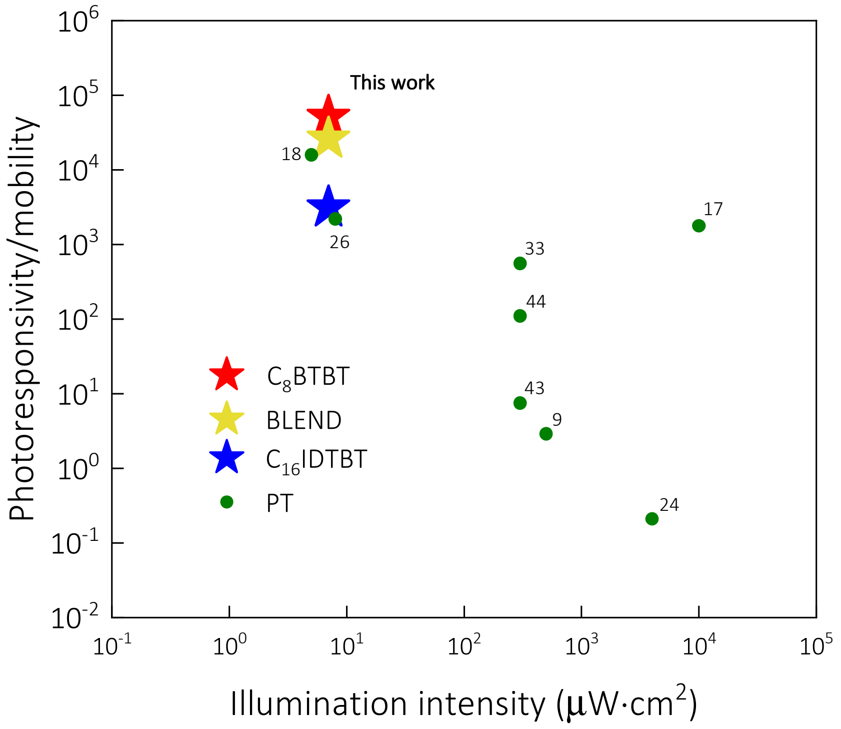

Supplementary Fig. 1. | Comparison of device performance reported in the literature. The numbers next to the data indicate the reference from which the values were taken. All the perovskite-based phototransistors listed here are phototransistor (PT) type devices that can operate in both active mode and switch mode. C_8-BTBT, BLEND (i.e. C_8-BTBT : C_{16}-IDTBT = 1:1) and C_{16}-IDTBT stand for the organic transistor channel materials used in the corresponding heterojunction phototransistors (HJPTs) reported in this work.

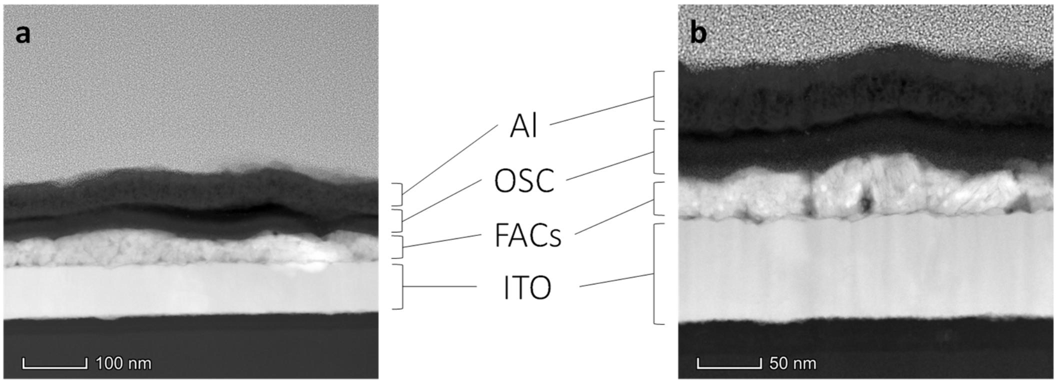

Supplementary Fig. 2. | HAADF-STEM characterisation for FACs/blend 1:1 film stack. HAADF-STEM characterization for FACs/blend 1:1 film stack. HAADF-STEM images for the FACs/blend 1:1 film stack deposited on ITO/glass: (a) low magnification; (b) high magnification.

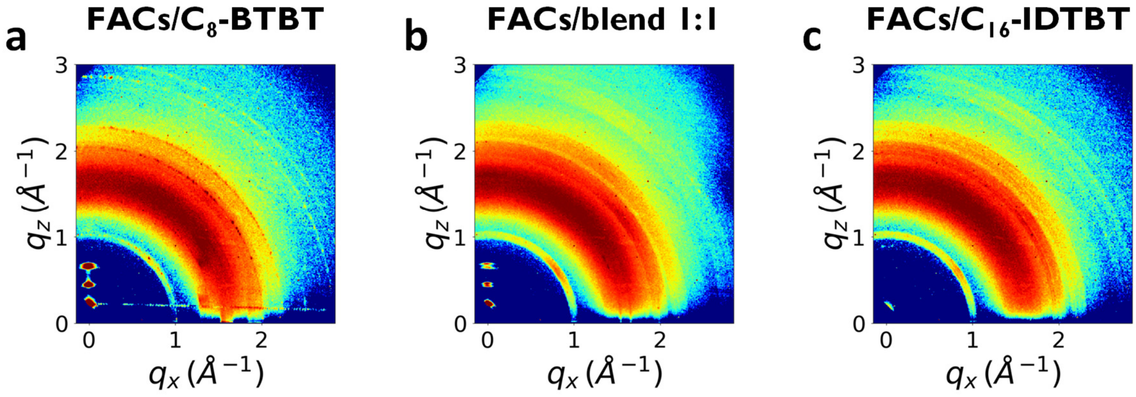

Supplementary Fig. 3. | GIWAXS patterns for FACs/OSC films at a low-incident angle. GIWAXS patterns for the FACs/OSC films with a X-ray incident angle of 0.05°. GIWAXS patterns of (**a**) FACs/C_8-BTBT; (**b**) FACs/Blend 1:1; (**c**) FACs/C_{16}-IDTBT.

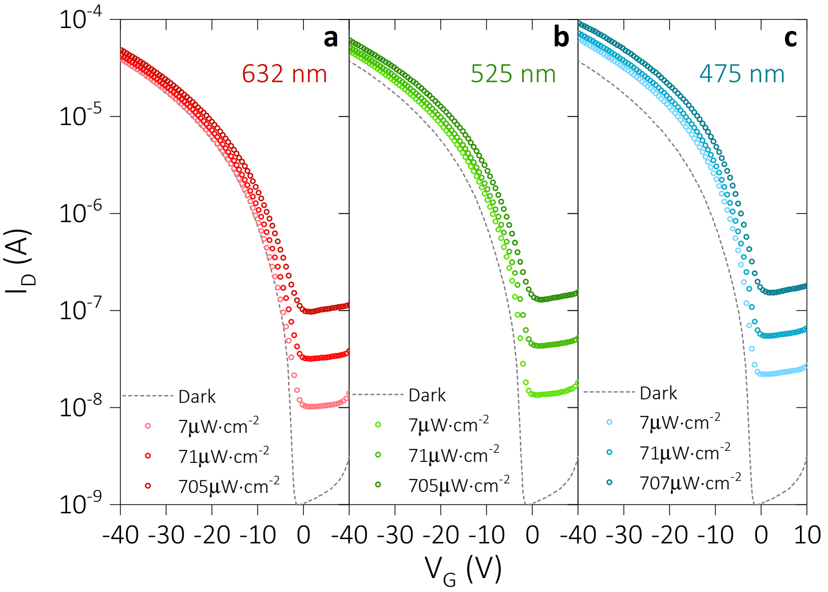

Supplementary Fig. 4. | Optoelectronic characterisation of HJPT with pristine C_8-BTBT. Transfer characteristics of a FACs/OSC heterostructure HJPT with C_8-BTBT only measured in dark and under illumination of (**a**) red light (λ_{peak}= 632 nm), (**b**) green light (λ_{peak}= 525 nm) and (**c**) blue light (λ_{peak}= 475 nm) with three different optical powers densities. The device was operated at a constant drain voltage (V_D) of -40 V

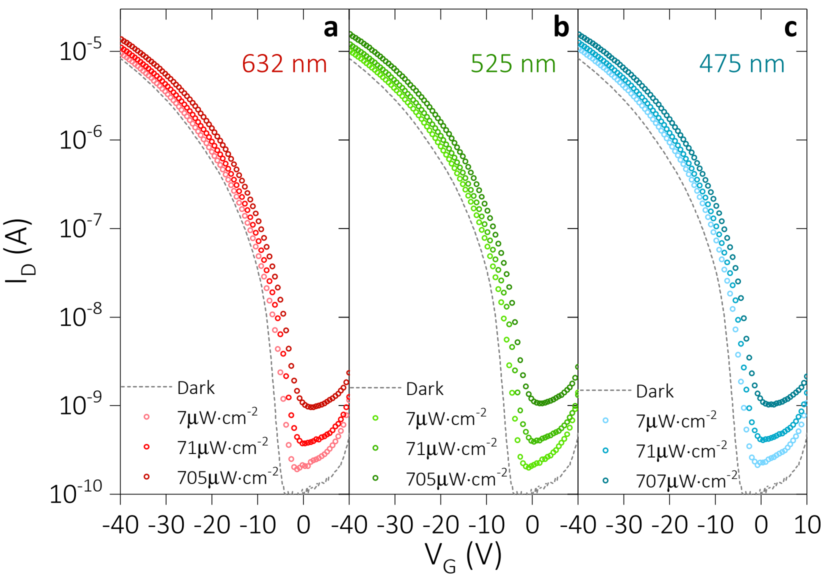

Supplementary Fig. 5. | Optoelectronic characterisation of HJPT with Blend 3:1. Transfer characteristics of a FACs/OSC heterostructure HJPT with a blend of C_8-BTBT and C_{16}-IDTBT in a ratio of 3:1 measured in dark and under illumination of (**a**) red light (λ_{peak}= 632 nm), (**b**) green light (λ_{peak}= 525 nm) and (**c**) blue light (λ_{peak}= 475 nm) with three different power densities. The device was operated at a constant drain voltage (V_D) of -40 V.

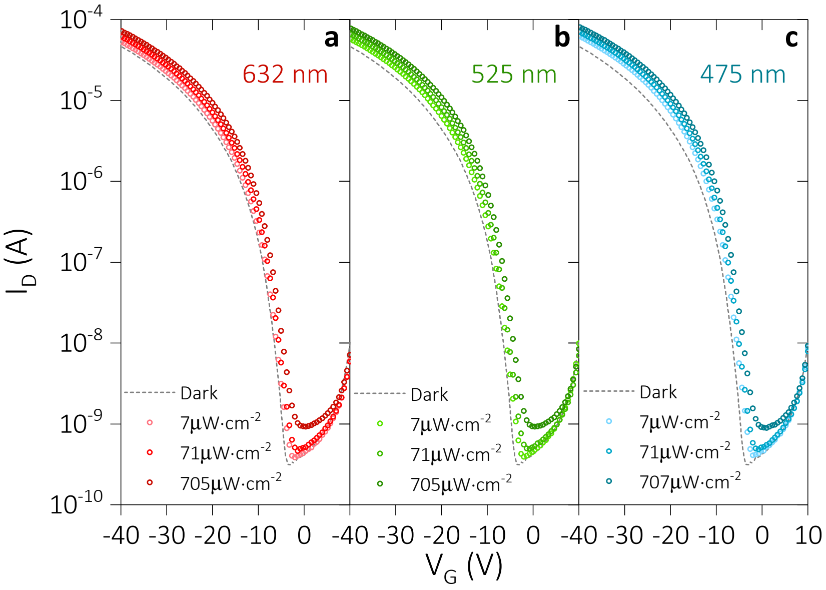

Supplementary Fig. 6. | Optoelectronic characterisation of HJPT with Blend 1:1. Transfer characteristics of a FACs/OSC heterostructure HJPT with a blend of C_8-BTBT and C_{16}-IDTBT in a ratio of 1:1 measured in dark and under illumination of (**a**) red light (λ_{peak}= 632 nm), (**b**) green light (λ_{peak}= 525 nm) and (**c**) blue light (λ_{peak}= 475 nm) with three different power densities. The device was operated at a constant drain voltage (V_D) of -40 V.

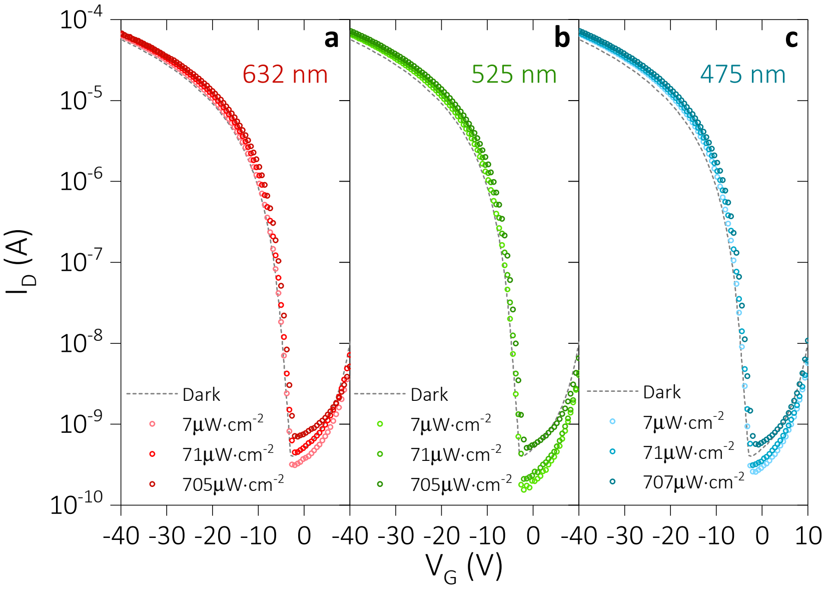

Supplementary Fig. 7. | Optoelectronic characterisation of HJPT with Blend 1:3. Transfer characteristics of a FACs/OSC heterostructure HJPT with a blend of C_8-BTBT and C_{16}-IDTBT in a ratio of 1:3 measured in dark and under illumination of (**a**) red light (λ_{peak}= 632 nm), (**b**) green light (λ_{peak}= 525 nm) and (**c**) blue light (λ_{peak}= 475 nm) with three different power densities. The device was operated at a constant drain voltage (V_D) of -40 V.

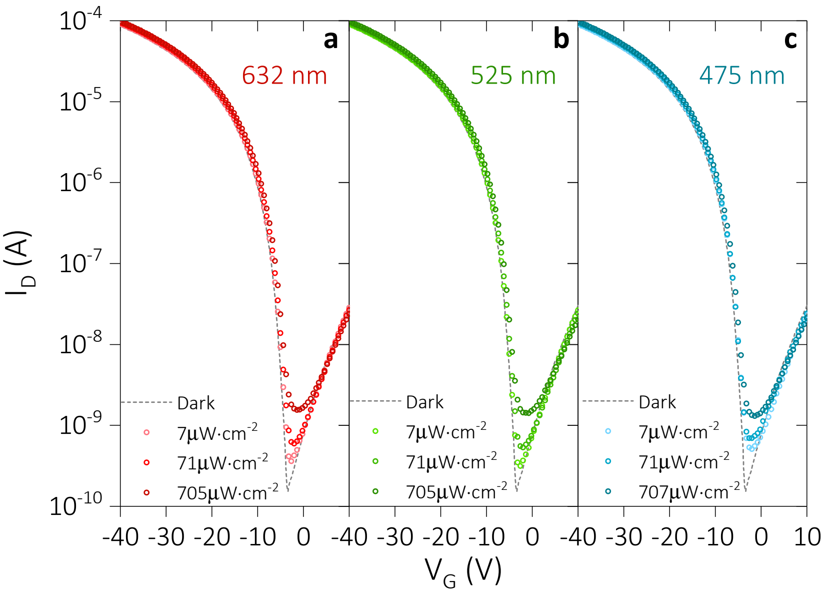

Supplementary Fig. 8. | Optoelectronic characterisation of HJPT with pristine C_{16}-IDTBT. Transfer characteristics of a FACs/OSC heterostructure HJPT with C_{16}-IDTBT only measured in dark and under illumination of (**a**) red light (λ_{peak}= 632 nm), (**b**) green light (λ_{peak}= 525 nm) and (**c**) blue light (λ_{peak}= 475 nm) with three different power densities. The device was operated at a constant drain voltage (V_D) of -40 V.

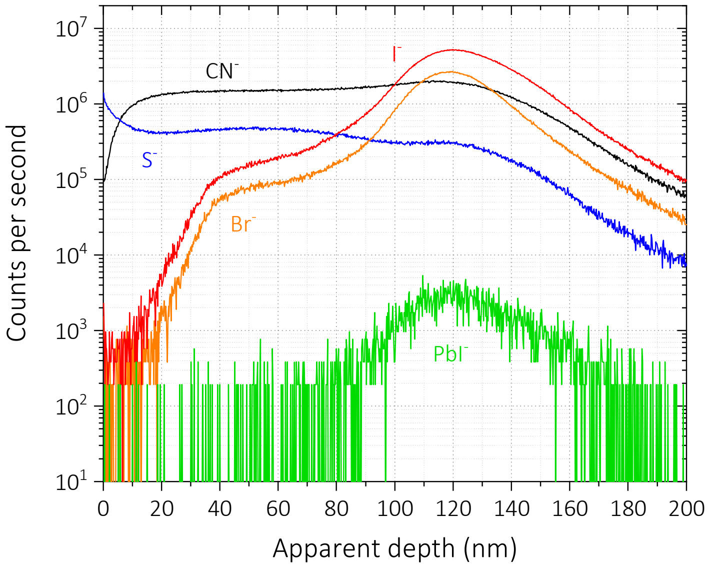

Supplementary Fig. 9. | Negative DSIMS depth profile for FACs/blend 1:1 film stack. Dynamic secondary ion mass spectrometry (DSIMS) characterisation performed for the FACs/blend 1:1 film stack.

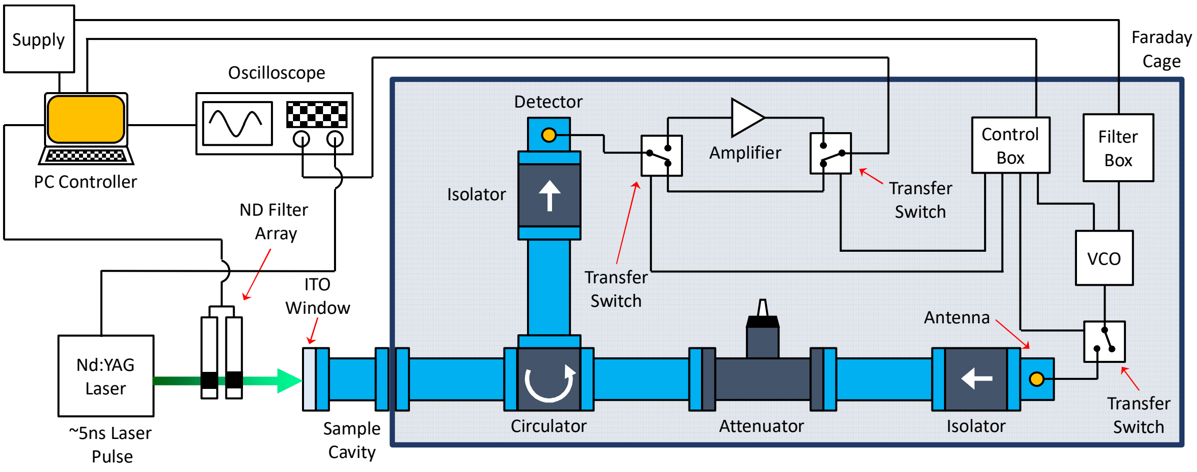

Supplementary Fig. 10. | Schematic representation of the TRMC system. A voltage-controlled oscillator (VCO) is used to generate a microwave-frequency (~8.5 GHz) electrical signal. The oscillatory signal is guided into an antenna that emits microwaves into the waveguide. After passing through an isolator and an attenuator, the microwaves enter a circulator, which acts as a unidirectional device: the signals entering from port 1 exit through port 2; and the signals entering from port 2 exit through port 3. The incident microwaves then pass through an iris, into a sample cavity, and form a standing wave. The test sample is placed into the cavity at one of the maxima of the standing wave. Free carriers within the sample absorb microwaves, resulting in a change in the reflected microwave signal. The latter exits through circulator and is then detected using a zero-bias Schottky diode. The resulting voltage is subsequently amplified, then measured using an oscilloscope. Photocarriers are excited using a pulsed Nd:YAG laser with emission frequency of 532 nm and full-width at half maximum (FWHM) of 5 ns. This schematic drawing is adapted with permission from Crovetto *et al.*, *Chem. Mater.* **31**, 3359-3369 (2019). Copyright 2019 American Chemical Society.

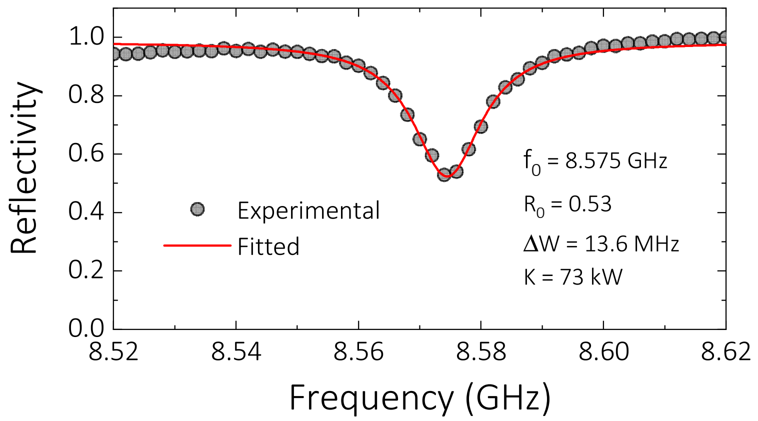

Supplementary Fig. 11. | Cavity response example. Example reflectivity of cavity measured as a function of frequency (points). The red line is a fit to **Supplementary Equation 4**, with fitting parameters of f_0 = 8.575 GHz, R_0 = 0.53, ΔW = 13.6 MHz.

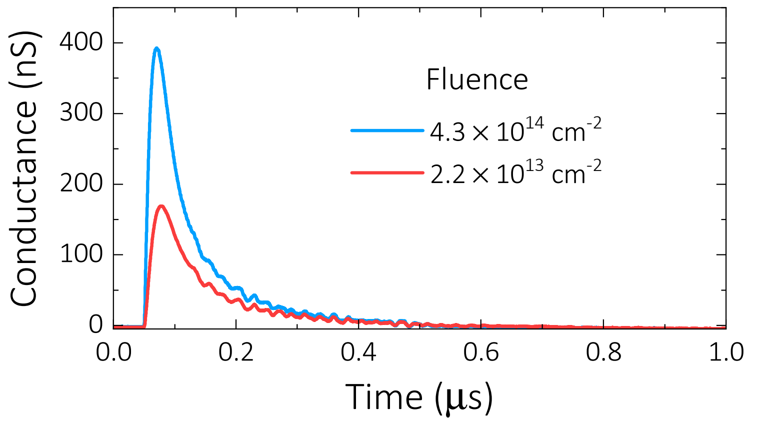

Supplementary Fig. 12. | Example of TRMC transient data. Change in conductance due to optical illumination as a function of time for a thin film of pristine perovskite on quartz, at two different optical fluences. The laser wavelength was λ = 532nm. Samples were measured at room temperature in air.

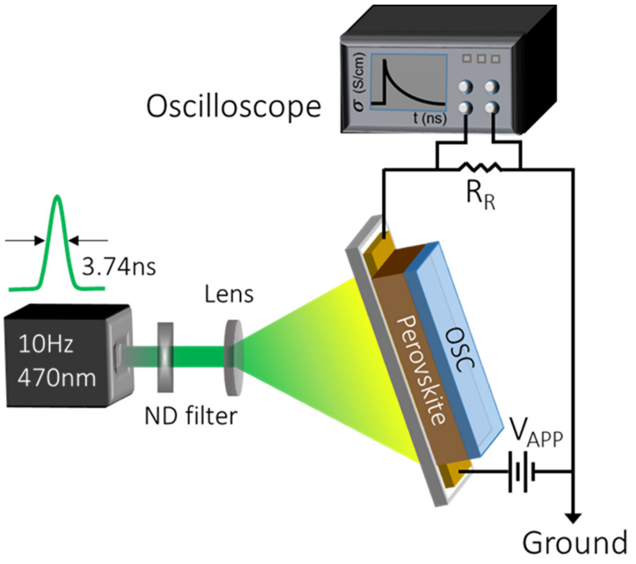

Supplementary Fig. 13. | Schematic representation of TPC measurement setup. A Nd:YAG pulsed laser excitation source pumped at 10 Hz with FWHM = 3.74 ns was set to 470 nm with a range of fluences in order to vary the charge carrier densities of FACs. A bias of 24 V is applied on one of the in-plane electrodes. A variable resistor is connected in series with the samples. The voltage drop on this resistor was monitored through an oscilloscope with a high internal resistance (1 MΩ) connected in parallel to determine the change of the potential across the two in-plane Au electrodes.

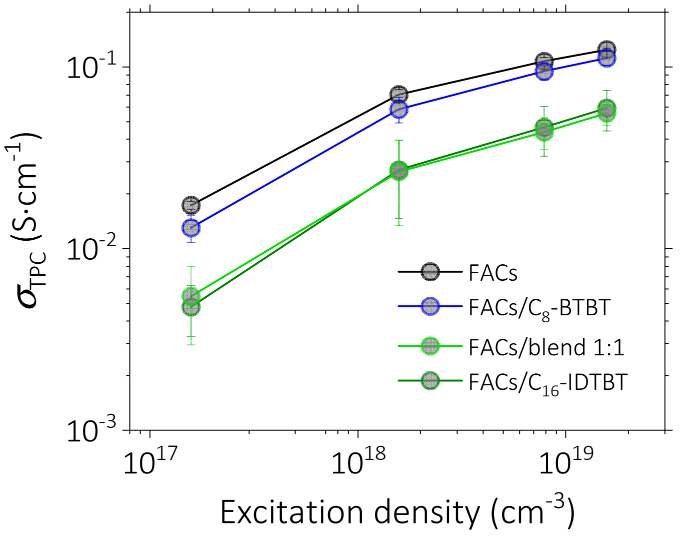

Supplementary Fig. 14. | TPC data for pristine FACs film and FACs/OSC bilayer stacks. TPC conductivity is plotted as a function of excitation density. The error bars denote standard deviations.

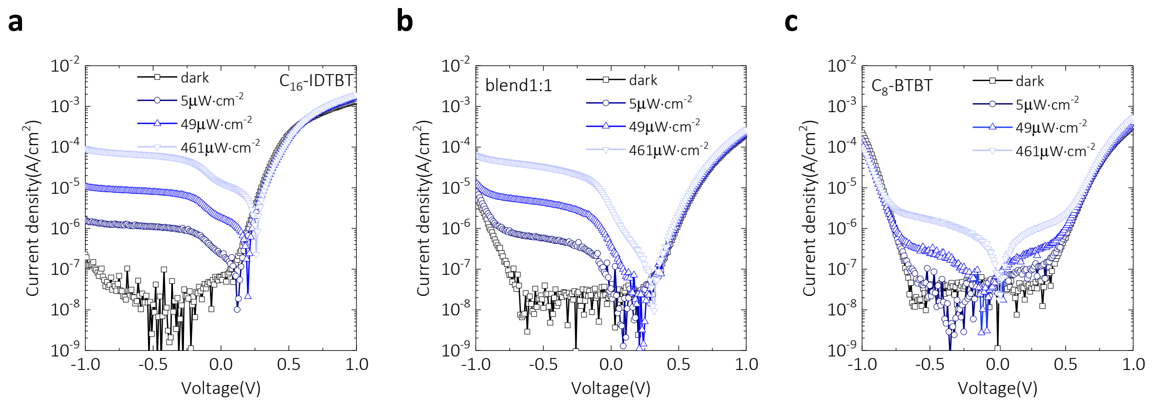

Supplementary Fig. 15. | Optoelectronic characterisation of perovskite-organic diodes. Current voltage characteristics of diodes based on FACs/OSC heterostructure measured in the dark and under different illumination intensities using a green LED light source (λ_{peak}= 525 nm): (**a**) FACs/C_8-BTBT, (**b**) FACs/Blend 1:1 and (**c**) FACs/C_{16}-IDTBT. The FACs/OSC stacks were sandwiched by two symmetric Au electrodes.

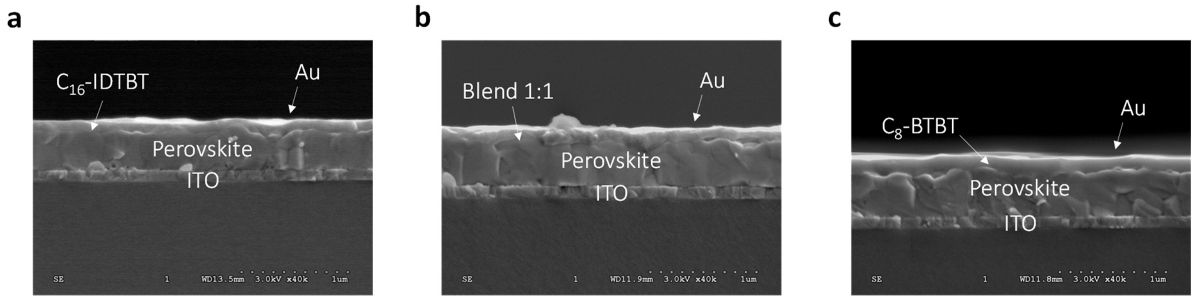

Supplementary Fig. 16. | SEM images for FACs/OSC bilayer stacks. SEM cross-sectional images for FACs/OSC bilayer stacks made on ITO/glass with Au top electrodes: (**a**) FACs/C_8-BTBT, (**b**) FACs/C_{16}-IDTBT and (**c**) FACs/blend 1:1.

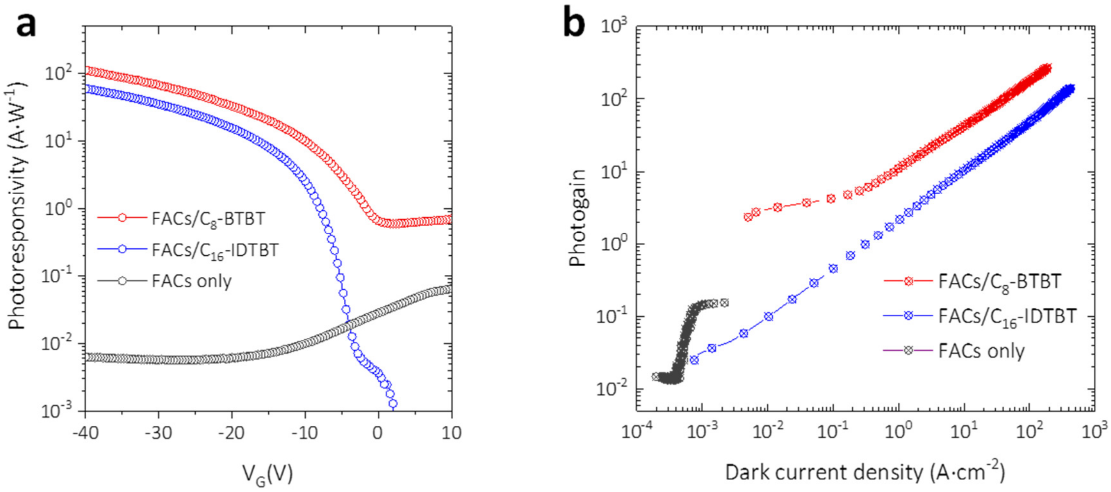

Supplementary Fig. 17. | Photodetection properties of FACs transistor and HJPTs. (**a**) Gate-voltage (V_G) dependent photoresponsivity and (**b**) dark-current dependent photogain measured at drain voltage V_D = -40 V for phototransistor based on pristine perovskite (i.e. FACs only) as well as three representative HJPTs using FACs/C_8-BTBT and FACs/C_{16}-IDTBT under the illumination at 532 nm & 500 μW·cm^{-2}.

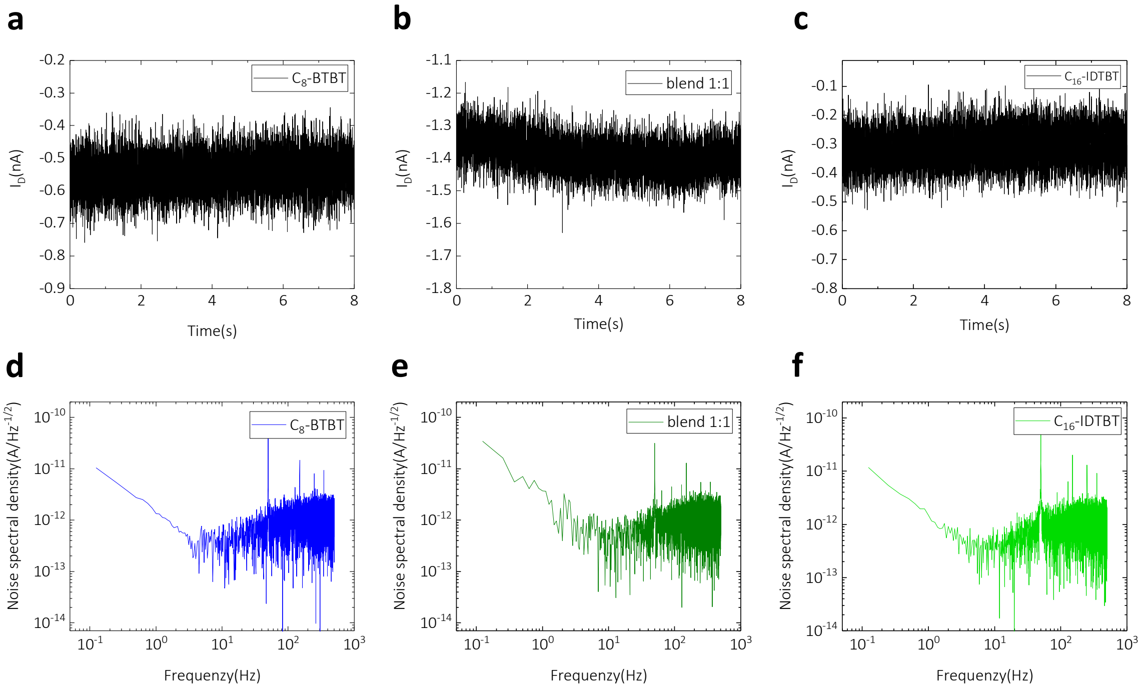

Supplementary Fig. 18. | Analysis of noise spectral density of FACs/OSC HJPTs. The dark current waveforms of FACs/OSC HJPTs with (**a**) pristine C_8-BTBT, (**b**) blend in a ratio of 1:1 and (**c**) pristine C_{16}-IDTBT measured at a constant drain voltage (V_D) of -40 V. (**d**),(**e**),(**f**), analysis of noise spectral density of the HJPTs based on the Fourier transform of dark current waveform shown in (**a**), (**b**), (**c**).

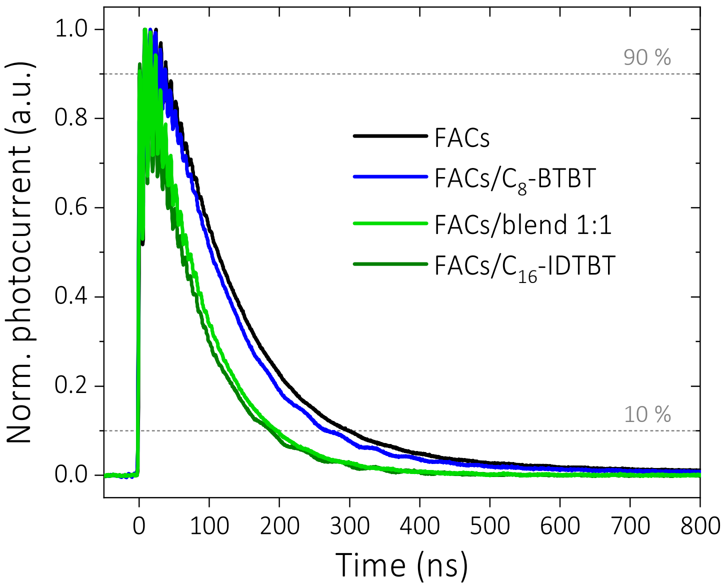

Supplementary Fig. 19. | TPC for studying HJPT response time. Photocurrent response for pristine FACs and various FACs/OSCs was studied via a transient photoconductivity measurement technique (see **Methods** and **Supplementary Figure 13**). The light source applied was a Nd:YAG pulsed laser (470 nm) with FWHM = 3.74 ns. The device dimension employed was 4 mm by 5 mm enclosed by two parallel Au electrodes (one with applied voltage of 24 V; the other one grounded).

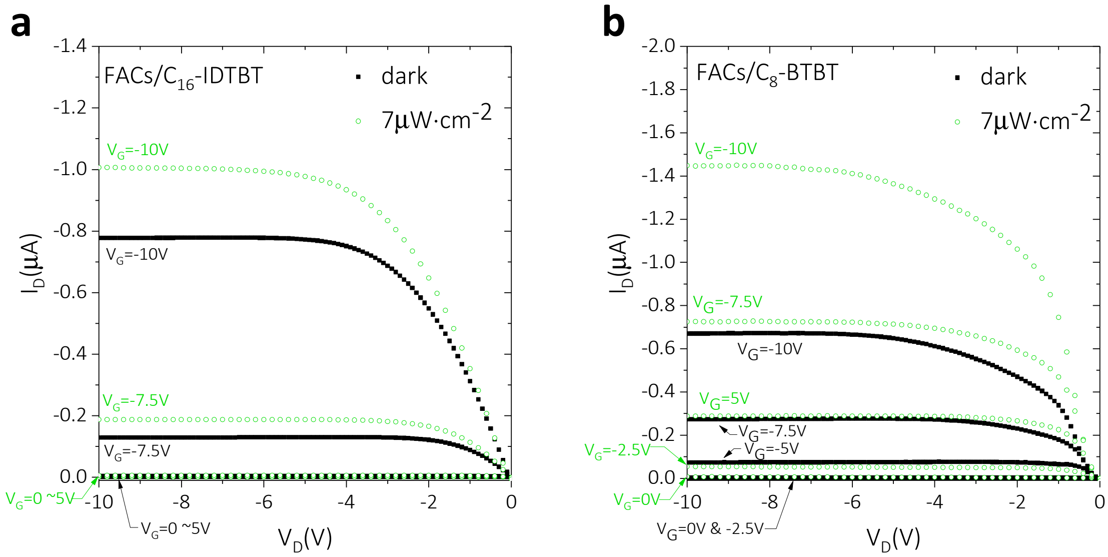

Supplementary Fig. 20. | Output characteristics of HJPTs. Output characteristics of FACs/OSC heterostructure HJPTs with (**a**) C_{16}-IDTBT only and (**b**) C_8-BTBT only measured in dark and under illumination of green light (λ_{peak}= 525 nm). The gate voltage (V_G) was varied from 0 V to -10 V in a step of 2.5 V.

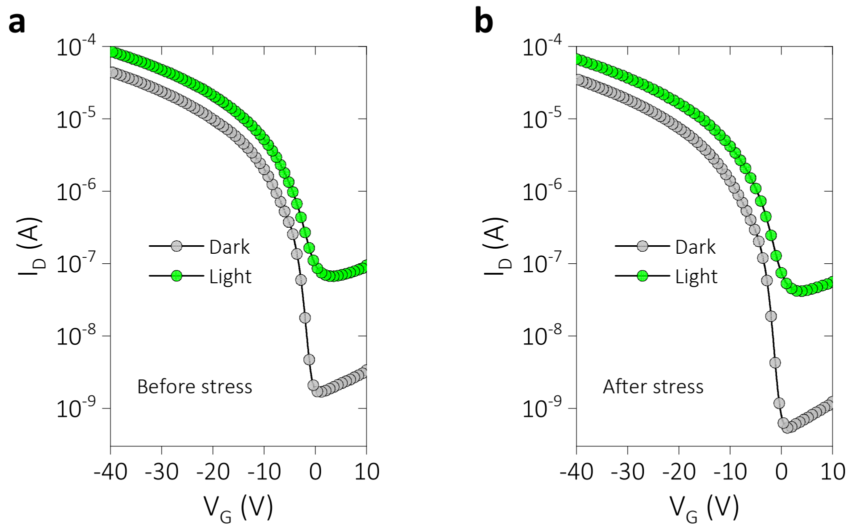

Supplementary Fig. 21. | Bias-stress stability for FACs/C_8-BTBT HJPTs. Transfer current-voltage characteristics (V_D = -10 V) for a FACs/C_8-BTBT HJPT under a bias-stress stability test with a test condition of applying constant V_G = -40 V and V_D = -10 V for a continuous period of 2×10^4 sec, simulating a transistor continuously operating in a working mode: (**a**) before and (**b**) after the bias-stress test. The illumination was carried out using a green LED light source (λ_{peak}= 525 nm) with an intensity of 705 $\mu W \cdot cm^{-2}$. The reason for testing the device only at its initial and final states is to avoid the potential redistribution of the bias-stress-induced trapped charge carriers from the light illumination[68-70], which could result in a less-rigorous, less-severe bias-stress condition. Our FACs/C_8-BTBT HJPT exhibits only a minor drain current deterioration (~20 % for I_{ON} measured in the dark) as well as a nearly negligible V_{ON} shift (ΔV_{ON} = ~0.1 V). Our demonstration represents an excellent example for high bias-stress stability for a C_8-BTBT based TFT[71]. Importantly, after the bias-stress test we have only observed minor deterioration (~21%) in the I_{ON} when measured under illumination.

Supplementary Tables

Supplementary Table 1. | Transistor performance of FACs/OSC HJPTs

Composition (C_8-BTBT:C_{16}-IDTBT)	Mobility ($cm^2V^{-1}s^{-1}$)	Threshold voltage V_{TH} (V)	Turn-on voltage V_{ON} (V)	On/off ratio	Subthreshold swing S.S. (dec/V)
1:0	0.33	-4.1	-1.6	2.5×10^3	9.4
3:1	0.05	-6.4	-4.0	2.0×10^3	6.5
1:1	0.42	-7.3	-3.6	4.3×10^3	3.5
1:3	0.57	-5.6	-2.8	9.2×10^3	4.1
0:1	1.02	-6.6	-3.4	2.4×10^4	4.8

Supplementary Table 2. | TRMC figure of merit.

**Sample**	**$\phi\Sigma\mu_{TRMC}$ (cm^2/Vs)**
FACs	0.8
FACs/C_8-BTBT	0.61
FACs/C_{16}-IDTBT	0.15
FACs/blend 1:1	0.2

Supplementary Table 3. | HJPTs figure of merit from current noise characterisation

composition	R(AW^{-1})	I_n(pA)	NEP($W\cdot Hz^{-1/2}$)	D*(Jones)
C_8-BTBT	1.7×10^4	1.3	7.8×10^{-17}	2.2×10^{14}
Blend 1:1	1.1×10^4	3.7	3.5×10^{-17}	4.9×10^{14}
C_{16}-IDTBT	3.2×10^3	1.5	4.5×10^{-16}	3.8×10^{13}

Supplementary Notes

Supplementary Note 1; General discussion on perovskite-based photodetector

Classification of photodetectors. Photodetectors can be classified in several ways in terms of materials, devices architecture and dominant mechanism[1–3]. From the perspective of input-output signal gain, two subcategories of photodetectors can be identified, i) photodiodes with no photogain; and ii) photoconductors/phototransistors with photogain[4]. In conventional photodiodes, an absorbed photon can at maximum generate one electron-hole pair, corresponding to an EQE of 100%. In this ideal circumstance, this type of devices can generate a maximum photogain of unity. In photoconductors/phototransistors, the metal-semiconductor contacts allow continuous injection of charge carriers, and photodetectors of this kind can result in a high EQE level that exceeds 100%[4]. The reason is because the majority carriers in a semiconductor can be fast collected by the drain electrode under the conditions: i) delayed recombination (e.g., due to the trapping of minority carriers); and ii) the majority charge carriers can be (re)injected from the metal contacts to achieve charge neutrality. The process can continue until the carriers recombine. Therefore, photocarriers with the same polarity as the majority carriers in a given semiconductor can add to the circulating current between the two electrodes (e.g. from the source to drain contacts). This, in short, means that upon illumination a photon can trigger a current flow generated by the majority carriers to circulate for many cycles when the transit time is shorter than the carrier lifetime, resulting in high photogain.

Difference between photoconductors and phototransistor. Photoconductors are usually constructed in a planar structure[4], where photocarriers are transported between two parallel metal electrodes. This planar structure avoids the strict requirements of film quality and electrodes transparency in the vertical counterpart and enables direct electrical property modulation by light. Phototransistors, on the other hand, have a gate electrode and a dielectric layer added to the planar structure, forming a three-terminal structure. The electrical property of this type of device is determined by mobile charge carriers in the semiconductor channel, which can be modulated by either gate bias or incident light. Compared to two-terminal devices, this three-terminal structure provides an extra degree of freedom for device operation and tuneable optoelectronic properties. Having said that, one should note that there are many reports that show phototransistor-like photoconductor devices (i.e., three metal terminals and a gate dielectric)[5–13]. However, these devices still behave like a photoconductor, rather than a

phototransistor, because there are certain criteria that need to be fulfilled. We will address this in the following paragraph.

Just identical to normal transistors, a phototransistor should possess two operation regimes, namely linear regime and saturation regime[14]. These two operation regimes determine two different operation modes in phototransistor circuit. In active mode, a phototransistor is working in linear regime and provide a response proportional to incident light intensity. In switch mode, a phototransistor operated in either cut-off regime or saturation regime exhibits different nonlinear current levels when varying illumination intensities. The non-linear response and saturation of channel current at high light intensity generate two output logic levels. Device working in this mode acts like a digital switch. The existence of such two operation modes distinguishes phototransistors from photoconductors in the aspect of circuit application.

Photogain/dark current trade-off in photoconductive devices. The photogain mechanism for photoconductors and phototransistors working in the photoconductive regime is usually described as photoconductive gain[15]. It is defined as the ratio of photogenerated carrier life time to the carrier transit time as described by **Equation 3** in **Method**. Photodetectors dominated by such mechanism are termed as photoconductive devices. Within these devices a high photogain is usually obtained using materials with high carrier mobility as the latter can result in a much shorter carrier transit time than carrier lifetime. However, these high mobility materials inevitably lead to a high level of current even when the devices are not exposed to light (i.e., high dark current). Such high dark current is undesirable for photodetectors in most of cases as it causes a high level of shot noise[16] and limits the detectivity of the devices (see **Supplementary Note 5** for detailed discussion on current noise). Therefore, a trade-off between photogain and dark current is commonly observed to exist in photoconductive devices.

Perovskite-based phototransistors. The simplest way to construct perovskite phototransistors is using a single-component (i.e., perovskites) for both light absorbing and charge transporting. In order to achieve high performance, this single perovskite thin-film layer should fulfil both high field effect mobility and high photoresponsivity. However, there have been only a few reports on this type of structures so far[9,17,18]. For example, Li *et al.*[17] reported a phototransistor based on a solution-processed polycrystalline $CH_3NH_3PbI_3$ film with photoresponsivity of 320 A W^{-1} and field-effect mobility of 0.1 cm^2 V^{-1} s^{-1} when the device was under strong illumination of 10 mW cm^{-2}. On the other hand, the performance of this phototransistor in the

dark is poor, exhibiting an extremely low drain current below 0.5 nA and small on/off ratio of less than one order of magnitude. These device performance parameters could largely limit its functionality as an electrical switch, especially in a low-light condition. The reason behind this could be attributed to strong defect recombination[19]. To address this and improve the device performance research efforts have been put on optimising crystalline structure as well as film morphology[9,18,20–22]. For example, Cho *et al.*[9] reported a perovskite phototransistor with an enhanced mobility of 2.1 cm^2 V^{-1} s^{-1} using an orientationally pure crystalline $CH_3NH_3PbI_3$ film with unique crystal orientation and low trap-state density. The enhancement in mobility is observed for charge transport along the crystal orientation. However, the resulting photoresponsivity is only 6.1 A W^{-1}.

As pointed out in the main text, adopting hybrid structure provides an alternative way to achieve high-performance phototransistors. In hybrid structure perovskites are often combined with other functional materials that possess excellent electronic transport properties[23–26]. Using this approach, the difficulty of getting a functioning perovskite-based transistor caused by the ionic movement in perovskite can also be mitigated[27,28]. 2D layered materials such as graphene[6–8,10–13,29–31] and transition-metal dichalcogenides (TMDs)[24,32–34] have been demonstrated to form an effective charge transport channel in a hybrid structure with perovskite materials. Among various transistor materials, graphene has been extensively studied due to its ultrahigh carrier mobility (~10^4 cm^2 V^{-1} s^{-1})[35,36]. The first graphene/perovskite heterojunction phototransistors was reported by Lee *et al.* and showed a photoresponsivity of 180 A W^{-1} with a solution-processed $CH_3NH_3PbI_3$ film[6]. The photoresponse in such graphene/perovskite structure is explained by a photogating effect that enhances photoconductive gain. To reduce bulk recombination in perovskite as well as to increase photoresponsivity, research efforts have been focused on micro/nanostructure engineering on perovskites layers. For example, Wang *et al.* reported a graphene/perovskite hybrid phototransistor by forming a thin layer of dispersive $CH_3NH_3PbI_3$ island on top of the graphene layer[7]. The resulting devices showed a high photoresponsivity of 6.0 × 10^5 A W^{-1}. Spina *et al.* employed $CH_3NH_3PbI_3$ nanowire in a heterostructure with graphene[8] and obtained a device with photoresponsivity as high as 2.6 × 10^6 A W^{-1}. Shao *et al.* covered a graphene monolayer with a 2D multiphase perovskite film[13], achieving a phototransistor with high photoresponsivity of ~10^5 A W^{-1}.

Although ultrahigh photoresponsivity can be achieved using a heterostructure composed of graphene/perovskite under extremely low illumination intensities at the level of nW~μW cm^{-2},[8,30,31] this might result in this type of devices more suitable for low light

photodetection. Nevertheless, the most important message here is that these hybrid approaches have demonstrated devices with enhanced field-effect mobilities and current-voltage modulation. In combination with perovskite materials, most of the devices exhibited photoresponsivity of >10^4 A W^{-1}, which is higher than that reported for single-crystalline silicon phototransistor[38].

Issues of phototransistor with highly conductive channel materials. Based on similar hybrid-structure design as aforementioned, several different attempts that combined perovskite with other high-mobility or high-conductivity materials (e.g., carbon nanotubes[39,40] and PEDOT:PSS[37]) have been reported. For these approaches, high photoresponsivities >10^4 A W^{-1} were achieved. Despite the promising values of photoresponsivity, devices based on such materials also induce several issues, which are not ideal for phototransistor operation. These conductor-like materials (e.g., graphene, metallic-like carbon nanotubes and PEDOT:PSS) possess high mobility at the expense of tuneability of channel conductivity[41], causing poor current-voltage modulation[42] and leading to a transistor channel that cannot be switched off. As a result, it is often observed a high level of dark current[29] and/or limited detectivity[6] from these approaches. More critically, devices based on conductor-like materials usually show no saturation for channel current. The latter constrains these devices to operate in active mode only. Therefore, we would like to stress the fact that a photoconductive device based on conductor-like materials should be termed as a photoconductor rather than a phototransistor, even if this device is configured in a three-terminal structure.

Our heterojunction phototransistors (HJPTs) exhibit high photoresponsivity when using organic materials with field-effect mobilities in the range of 0.3 to 1 cm^2 V^{-1} s^{-1}. This result is particularly of interest as it demonstrates a new route towards high photoresponsivity. From **Equation 1** (see **Methods**), photoresponsivity is defined as the ratio of photogenerated current to incident optical power. As such, increasing the level of photocurrent is the key to obtain high photoresponsivity. The concept of using high-mobility materials is to enlarge photocurrent by enhancing charge carrier circulation in a transistor channel for a given number of photogenerated charge carriers. In this scenario, devices with moderate mobility should only exhibit moderate photoresponsivity if there are the same number of photogenerated carriers involved in this circulation process. The demonstration of high values of photoresponsivity using our approach indicates that the enlargement in photocurrent is attributed to other factors, rather than enhanced photocarrier circulation through high-conductivity materials. Instead, this improvement is attributed to improved efficiency of preserving photocarriers that are involved

in carrier circulation. This process is realised by engineering a type-I straddling MHP/OSC heterojunction. To enable a direct comparison between phototransistors with different mobilities, a normalised photoresponse is proposed here by calculating the quotient of photoresponsivity and mobility. The comparison of phototransistor performance of our work and other perovskite-based phototransistors is then performed by plotting the normalised photoresponse as a function of illumination intensity (**Supplementary Figure 1**). Our work has demonstrated one of the highest normalised photoresponse values among the reported phototransistors (i.e. devices capable of operating in both active mode and switch mode)[9,17,18,24,26,33,43,44]. This value is even found to be comparable to those of gate-tuneable photoconductors[11,30,31,34,40,45].

Supplementary Note 2; Optoelectronic characterisations for HJPTs

Supplementary Figures 4-8 show optoelectronic properties for FACs HJPTs with different OSC compositions, i.e. pristine C_8-BTBT, three C_8-BTBT:C_{16}-IDTBT blends (3:1, 1:1 and 1:3) and pristine C_{16}-IDTBT, measured in the dark and under illuminations of red, green and blue light with various optical power densities. In **Supplementary Table 1**, we summarise key transistor performance parameters for all the HJPTs when measured in the dark. Under illumination, the increase in I_D was observed universally in all conditions, and the increment of I_D is proportional to the incident power density.

In the C_8-BTBT based HJPT, the photoresponse appear to consist of two components, i.e. transistors turned on at more positive V_G and a significant increase in the I_D at the off-state. Especially, I_D at the off-state was increased by an order of magnitude even under the illumination of the lowest power density. The observation of a more positive turn-on voltage (V_{ON}) is attributed to a change in the flatband voltage caused by the photogenerated carriers accumulated at the back channel side[46]. This phenomenon has been confirmed by our spectroscopic studies (**Figure 3**) and Mott-Schottky analysis (**Figure 4**). Moreover, the fact that an apparent increase was observed in the off-current is evidencing the back-channel charge accumulation. Similar to pristine C_8-BTBT, the blend 3:1 HJPT shows photoresponse consisting of a positive shift of V_{ON} and an increased I_D at the off-state. However, the increment in I_D at the off-state is less significant as a maximum increase of one order of magnitude was observed under the highest incident power density of 705 μW cm^{-2}.

In the blend 1:1 HJPT, no significant increase in I_D at the off-state is observed and the photoresponse can be mainly recognised by a shift of V_{ON} toward the positive V_G. This

photoresponse is close to the phenomenon described in an ideal hybrid phototransistor consisting of a channel semiconductor and a light absorber[47]. Such a change in photoresponse indicates that the photogating effect[48] becomes dominant with the increasing amount of C_{16}-IDTBT. In the blend 1:3 HJPT and the pristine C_{16}-IDTBT HJPT, the I_D modulation by light illumination becomes less prominent, suggesting poor photogenerated carrier circulation in the C_{16}-IDTBT dominant devices. This inefficient photocarrier circulation in the C_{16}-IDTBT HJPT could be attributed to the following two plausible mechanisms: one is nonradiative recombination losses at the FACs/OSC interfaces[49]; the other is mobile ionic defects in the FACs redistributing the electrostatic potential and screening the effect of photoelectron accumulation within the perovskite film[50]. Moreover, the injected carriers by applying large gate bias at transistor on-state is generally several orders of magnitude higher than the photoinduced carriers. This explains why the magnitude of the increased current levels (i.e., photoresponse) at transistor on-state is found to be less prominent compared to that in the off-state for all the HJPTs.

The photoresponse of all these five types of HJPTs also showed different wavelength dependence. In the C_8-BTBT based HJPT a strong wavelength dependence was observed, with a more significant photoresponse under blue illumination and a less significant photoresponse under red illumination. This strong wavelength dependence of photoresponse could be attributed to the higher absorption coefficient of FACs film at shorter wavelengths (as shown in **Figure 1c**). In contrast, the wavelength dependence becomes less prominent with the increasing amount of C_{16}-IDTBT. Such difference in wavelength dependence again confirms the inefficiency of photogenerated carriers transport and circulation in the C_{16}-IDTBT dominated channel.

Supplementary Note 3; Evaluation of time-resolved microwave conductivity figure of merit

A schematic representation of the time-resolved microwave conductivity (TRMC) system employed in this study is shown in **Supplementary Figure 10**. The microwave detectors employed in our system output a voltage (V) that is linearly proportional to the incident microwave power (*P*). The change in sample conductance due to optical illumination ΔG, is related to the normalized change in microwave power ($\Delta P/P$) via **Supplementary Equation 1**[51–53]:

$$\Delta G = -\frac{1}{K}\frac{\Delta P}{P} \tag{1}$$

where K is the sensitivity factor of the microwave cavity. Since ΔG is dependent on the normalized change in microwave power ΔP/P, it is not necessary to know the absolute values of microwave power, just the relative change under illumination. For this reason, with a knowledge of the detector voltage in the dark (i.e, V), and the change in detector voltage under illumination (i.e., ΔV), it is possible to determine the photo-induced conductance from **Supplementary Equation 1**. For the sensitivity factor of the microwave cavity (K), it can be evaluated from **Supplementary Equation 2**[52–54]:

$$K = \frac{2Q\left(1 + \frac{1}{\sqrt{R_0}}\right)}{\pi c \varepsilon_0 \varepsilon_r L \beta} \tag{2}$$

The parameters in **Supplementary Equation 2** are as follows: R_0 is the reflectivity of the cavity to microwaves at the resonance frequency f_0. L is the length of the cavity, where the length corresponds to the dimension in the direction of propagation of the electromagnetic waves. β is the ratio of the two remaining cavity dimensions, with the numerator/denominator depending on the polarisation direction of the standing wave. In our case, $\beta = 2.25$. ε_0 and ε_r are the vacuum permittivity and the relative permittivity of the medium of the cavity. Since our measurements are carried out in air, and the sample thickness is ~100 nm, and the cavity length is $L = 7.62$ cm we have here approximated $\varepsilon_r = 1$. Q is the quality factor of the cavity. This is the ratio of the resonance frequency of the full-width at half maximum of the cavity reflectivity resonance curve, as expressed in **Supplementary Equation 3**:

$$Q = -\frac{f_0}{\Delta W} \tag{3}$$

The parameter K can be evaluated by measuring the reflectivity of the cavity as a function of frequency, and fitting the response to a Lorentzian function[52], as expressed by **Supplementary Equation 4**:

$$R(f) = \frac{R_0 + \left(\frac{2(f - f_0)}{\Delta W}\right)^2}{1 + \left(\frac{2(f - f_0)}{\Delta W}\right)^2} \tag{4}$$

An example cavity response is shown in **Supplementary Figure 11**. By fitting **Supplementary Equation 4** to this data we are able to approximate the parameters f_0, R_0, ΔW, and hence K. For this example a sensitivity factor of 73 kΩ was measured.

During a TRMC experiment, ΔV is measured as function of time, before and after the sample is hit with a pulse (~5 ns) from an optical laser (λ = 532nm). With knowledge of V and K, one can then evaluate the photo-induced conductance (ΔG) as a function of time. **Supplementary Figure 12** shows some example data from a film of pristine perovskite on quartz. The peak observed value of ΔG (ΔG_{max}) can then be used to determine the TRMC figure of merit through **Supplementary Equation 5**[52,53]:

$$\phi\Sigma\mu_{\mathrm{TRMC}} = \frac{\Delta G_{\mathrm{max}}}{\beta e I_0 F_A M} \quad (5)$$

Supplementary Equation 5 is used to determine $\phi\Sigma\mu_{\mathrm{TRMC}} = \phi(\mu_e + \mu_h)$, where ϕ is the carrier generation efficiency, μ_e is the electron mobility in the semiconductor, μ_h is the hole mobility in the semiconductor, e is the magnitude of the fundamental unit of charge, I_0 is the fluence of the incident light and F_A is the fractional absorption of photons of the sample at the excitation wavelength (between 0 and 1). I_0 can be measured by placing a calibrated photodiode / thermopile in the path of the excitation path. F_A can be measured using ultraviolet–visible spectroscopy. M is a parameter we define as the "*masking parameter*" and is the fraction (between 0 and 1) of the cross-sectional area of the cavity that is exposed to the incident light.

Supplementary Equation 5 assumes that no recombination takes place at times < response-time of the measurement. The characteristic response time of the cavity (RC time) can be approximated via **Supplementary Equation 6**, and for our system is ~20 ns.

$$\tau_{RC} = \frac{1}{\pi \Delta W} \quad (6)$$

At low fluence this is a reasonable assumption, but at high fluence the carrier density is very high[55]. Under these conditions, bimolecular and Auger recombination can reduce the peak value of ΔG from what one would expect under ideal conditions[56]. This is manifest as a reduction in $\phi\Sigma\mu_{\mathrm{TRMC}}$ as a function of fluence at high fluence (as observed in **Figure 3c** of the main text). Using a simple model based on recombination during the finite duration of the laser pulse, this behaviour can be modelled. Specific details of the model can be found elsewhere[56],

but upon its application to our data we extract the representative values of $\phi\Sigma\mu_{\mathrm{TRMC}}$ as summarised in **Supplementary Table 2**.

Supplementary Note 4; Perovskite-organic diodes

To gain insight into the photogenerated charge transport in the FACs/OSC heterointerface, we fabricated three types of FACs/OSC heterojunction diodes with different OSC compositions including pristine C_{16}-IDTBT, pristine C_8-BTBT and organic blend in a ratio of 1:1. These diodes were constructed by sandwiching the FACs/OSC bilayer stacks by two Au electrodes with a thickness of 20 nm. The optoelectronic characterisation was carried out by applying a voltage sweep to the Au electrode next to the OSC and grounding the Au electrode in contact with the FACs to acquire the current-voltage characteristics in the dark and under green illumination (λ_{peak} = 525 nm) of various intensities. The semi-transparent Au electrode resulted in a 55% loss of incident optical power, which was measured separately by a UV-Vis instrument and a power meter. The obtained current-voltage (I-V) characteristics are shown in **Supplementary Figure 15**.

For both the FACs/C_{16}-IDTBT and FACs/blend 1:1 devices, typical diode I-V characteristics are observed in the dark (Supplementary Figures 15a and 15b) whilst upon light illumination, significant photoresponses arise in the diode saturation current region. The rectifying I-V curves and the corresponding photoresponses further corroborate the formation of the type-II heterojunction between FACs and C_{16}-IDTBT. On the other hand, the FACs/C_8-BTBT device exhibits less-rectifying I-V curves in the dark, signifying the formation of a bilayer dielectric stack rather than a diode (Supplementary Figure 15c). Upon light illumination the symmetric I-V curve persists with a rising current level under both positive and negative bias conditions. Again, this is further evidence of the straddling type-I heterojunction that forms the non-rectifying junction between the HOMO level of C_8-BTBT and the VB edge of FACs.

Supplementary Note 5; Discussion on current noise

Relationship between dark current and noise level. Dark current plays an important role in determining the overall performance of photodetectors as it sets the sensitivity of a photodetectors by being a major source of electronic noise[1]. To quantify the sensitivity of a photodetector limited by noise, noise-equivalent power (NEP), which corresponds to the incident root mean square power required to produce a signal-to-noise ratio (SNR) of unity in a 1 Hz bandwidth, is a commonly used a figure of merit. It can be expressed as:

$$NEP = \frac{S_{noise}}{R} \quad (7)$$

where S_{noise} is noise spectral density and R is photoresponsivity. The sources of electronic noise can be explained by many mechanisms such as thermal noise, dielectric noise or flicker noise[4]. Since all sources of noises are independent, their contributions to the total noise spectral are additive. However, a commonly-accepted assumption is that the shot noise from dark current is the dominant contribution[16]. Based on this assumption, the noise spectral density can be approximated as:

$$S_{noise} = (2qI_{dark})^{1/2} \quad (8)$$

where q is electron charge and I_{dark} is dark current of photodetectors.

Characterisation of current noise in HJPTs. The shot-noise-based assumption provides a simple way to estimate the noise spectrum density but it might also lead to a severe underestimation[2,57]. The noise spectral density in this study therefore was obtained following a previously-reported approach[58]. This approach calculates the noise spectrum density based on Fourier analysis on the acquired device dark current waveform. In our work, the dark current waveforms of HJPTs with different OSC compositions were recorded when the devices were biased at a fixed V_D of -40V. The waveforms acquired in a sampling rate of 1 kHz are shown in **Supplementary Figures 18a-c**. By applying a Fourier transform to the dark current waveform, the noise spectral density was obtained as shown in **Supplementary Figures 18d-f**. The corresponding *NEP* can then be calculated via **Supplementary Equation 7** and the specific detectivity (D^*) can be obtained via **Equation 4**. The calculated *NEP* and D^* values at a modulation frequency of 1 Hz for the devices with different OSC compositions are summarised in **Supplementary Table 3**. We note that the responsivity of our HJPTs under the illumination of three LED light sources (λ_{peak}= 475, 525, 632 nm) can reach ~10^4 A W^{-1}, which corresponds to a *NEP* of 10^{-17} W $Hz^{-1/2}$ and a D^* of 10^{14} Jones, such high D^* value is two orders of magnitude higher than the value for Si photodetectors[16].

Supplementary Note 6; Discussion on response time and its limitation

Differences in response time for perovskite-based phototransistors. Fast response to optical signal is critical for optoelectronic devices as it represents the efficiency of charge transport and collection within the devices. The response speed of photodetectors is usually characterised by response time. In general, intrinsic properties of a material, such as defect states, can

significantly affect the photogenerated carrier transition during the excitation and relaxation process, resulting in huge difference in response time between various materials[5,59,60]. When it comes to phototransistors, the complexity in device structures and the existence of multiple interfaces introduce extra factors that might significantly influence how photocarriers' transition happens. As such, response time is found to vary from device to device even with the same photoactive and transport materials. Taking hybrid phototransistors based on graphene and $CH_3NH_3PbI_3$ as examples, Lee *et al.* reported a bottom-gate bottom-contact (BG-BC) phototransistor based on a graphene/$CH_3NH_3PbI_3$ heterostructure with a response time of ~600 ms[6]. Dang *et al.* reported a graphene field-effect transistor (GFET) in a bottom-gate top-contact (BG-TC) configuration with a layer of $CH_3NH_3PbI_3$ deposited on top of the GFET, and the completed device exhibited a response time of 5 s[10]. Spina *et al.*[8] reported a similar BG-TC approach to the work by Dang *et al.*[10], but instead of using $CH_3NH_3PbI_3$ films the absorber was composed of $CH_3NH_3PbI_3$ nanowires. The resulting phototransistor exhibited a response time of 80 s. The difference in response time ranging from milliseconds to seconds for hybrid phototransistors based on the same graphene/$CH_3NH_3PbI_3$ combination is apparently influenced by other factors that are beyond materials' intrinsic properties, such as parasitic capacitances due to different phototransistor architectures and connection resistance due to non-standardised measurement setups.

Study of response time in HJPT via transient photoconductivity. The main task of this work is to elucidate how to efficiently transfer, transport and circulate photocarriers in a hybrid perovskite/organic based heterostructure, hence providing the design concept of the light absorber and the transport layer in a hybrid phototransistor system. Likewise, to understand what kind of a role the fundamental material property plays in the context of response time is critical to adopt our HJPT concept for system-level integration and engineering.

In this section, transient photoconductivity (TPC, **Supplementary Figure 13**) was used to characterise how fast photogenerated carriers respond to an ultra-short light-pulse stimulation[61,62]. Photocarriers induced by the input laser pulse (FWHM = 3.74 ns) were collected by applying an in-plane voltage bias of 24 V to one electrode whilst keeping the other one grounded. The obtained results represent how long it takes for photocarriers to respond to illumination and to dissipate in the dark under the presence of an applied voltage using a setup that resembles transistor-like operation. **Supplementary Figure 19** shows the normalised photocurrent measured for pristine FACs and three different FACs/OSC combinations using our TPC setup. The fast rise time and slow fall time resulted in all the detected photocurrent

present in a distorted Gaussian-like shape with significant difference in fall time levels for pristine FACs and FACs/OSCs. The reason for the fast rise time in all samples could be attributed to a rather fast trap-filling process, which has been reported in various material systems[5,37,59,63].On the other hand, the slow fall time usually considered as the main limiting factor could be related to different photocarrier lifetime and/or a longer trap-release process[5,37,59,63]. The latter generally poses a serious challenge to mitigate the issue that causes slow response time.

Clearly, pristine FACs and FACs/C_8-BTBT exhibit a longer fall time than that of FACs/blend 1:1 and FACs/C_{16}-IDTBT. This result reflects the fact that photocarriers have been taken away from FACs when FACs is in contact with C_{16}-IDTBT as evidently shown in all the spectroscopic measurement carried out in this work (**Figure 3**). In this scenario, response time could be influenced by many factors, such as the mobility of OSCs and the built-in potential between FACs/OSCs. In the case of FACs/C_8-BTBT, since the majority of photocarriers remain at the heterointerfaces of FACs and C_8-BTBT, the photocurrent decay time of FACs/C_8-BTBT in **Supplementary Figure 19** is similar to that of pristine FACs. As such, even for a HJPT device with large dimension of 4 mm by 5 mm (see **Methods** for transient photoconductivity), the ideal response time for the transistor to be turned off (i.e., to dissipate trapped photocarriers) can reach ~200 ns (i.e. the fall time for FACs and FACs/C_8-BTBT in **Supplementary Figure 19**).

As a final remark to conclude the section of response time, we would like to point out an often-used approach to reduce slow fall time is to input a short pulse to neutralise trapped charge carriers before turning a phototransistor off[5,63]. This could however increase the complexity of device operation. Otherwise, to truly achieve fast response time research focus will inevitably involve the study of advanced process engineering, such as a self-aligned gate[64] to reduce parasitic capacitances and a shrinking device dimension to reduce carrier transit time[65]. Therefore, engineering a fast-speed device is a topic beyond the scope of this work, which is mainly focusing on understanding device physics.

Supplementary Note 7 | Discussion on opto-electronic circuit demonstration

Limits of phototransistor evaluation based on traditional assessing metrics. Phototransistors performance is usually evaluated through the performance metrics such as photoresponsivity, photogain and detectivity. These figures of merit characterise the efficiency of photodetectors related to the process of photon conversion into electron. However, being an

active device, phototransistor enables not only photodetection but also controllable signal amplification. Unique properties in phototransistors such as tuneability of photoresponse and current/voltage amplification cannot be assessed through those performance metrics set for traditional two terminal photodetectors. Therefore, a more comprehensive evaluation method that covers all aspects of the phototransistor as active photodetector is needed. Here we propose a simple circuit demonstration, i.e., a photo-inverter that could meet the requirements.

Phototransistor performance evaluation based on photo-inverter circuit. Similar to normal PMOS inverters, the operation of the proposed photo-inverter can be simply explained by a voltage divider model[66]. The resistance difference between constituent phototransistors determines the voltages dropped on HJPT-1 and HJPT-2, hence changing the level of V_{OUT}. The resistance of the depletion load HJPT-1 is controlled by the illumination intensity while for the electrical switch HJPT-2 this resistance is controlled by V_{IN}. To fulfil the function of voltage inversion, HJPT-1 and HJPT-2 needs to possess pronounced photoresponse and good current-voltage modulation, respectively. Since the proposed photo-inverter configiration is composed of two identical HJPTs, the function of input signal inversion with high voltage amplification A_i in the circuit can only be realised by devices with both good electrical switching and high photoresponse. In this regard, this circuit demonstration can be seen as a unique method for evaluating the performance of phototransistors.

Relationship between A_i and transistor electrical characteristics. The voltage amplification A_i, is defined as the slope of the inverter transfer characteristic (**Figures 6b** and **6c**). The maximum voltage amplication $A_{i,max}$, corresponding to the point with the sharpest V_{OUT} voltage swing, means that a small change in V_{IN} can result in a large variation in V_{OUT} during a switching transient. From the viewpoint of microelectronic circuit, $A_{i,max}$ is usually obtained when the two constituent transistors are simultaneously working in the saturation regions[66]. This figure of merit therefore can be used to distinguish phototransistors from photoconductors as high A_i value is unattainable for photoconductive devices due to the absence of saturation in channel current[67]. In contrast, the high $A_{i,\,max}$ of ~15 obtained in our photo-inverter can be attributed to the good output characteristics of HJPTs as shown in **Supplementary Figure 20.**

Supplementary References

1. Baeg, K.-J., Binda, M., Natali, D., Caironi, M. & Noh, Y.-Y. Organic Light Detectors: Photodiodes and Phototransistors. *Adv. Mater.* **25**, 4267–4295 (2013).

2. García de Arquer, F. P., Armin, A., Meredith, P. & Sargent, E. H. Solution-processed semiconductors for next-generation photodetectors. *Nat. Rev. Mater.* **2**, 16100 (2017).

3. Wang, H. & Kim, D. H. Perovskite-based photodetectors: Materials and devices. *Chem. Soc. Rev.* **46**, 5204–5236 (2017).

4. Sze, S. M. & Ng, K. K. *Physics of Semiconductor Devices, 3rd Edition. Wiley* (2006).

5. Konstantatos, G. *et al.* Hybrid graphene–quantum dot phototransistors with ultrahigh gain. *Nat. Nanotechnol.* **7**, 363–368 (2012).

6. Lee, Y. *et al.* High-performance perovskite-graphene hybrid photodetector. *Adv. Mater.* **27**, 41–46 (2015).

7. Wang, Y. *et al.* Hybrid Graphene-Perovskite Phototransistors with Ultrahigh Responsivity and Gain. *Adv. Opt. Mater.* **3**, 1389–1396 (2015).

8. Spina, M. *et al.* Microengineered CH3NH3PbI3 Nanowire/Graphene Phototransistor for Low-Intensity Light Detection at Room Temperature. *Small* **11**, 4824–4828 (2015).

9. Cho, N. *et al.* Pure crystal orientation and anisotropic charge transport in large-area hybrid perovskite films. *Nat. Commun.* **7**, 1–11 (2016).

10. Dang, V. Q. *et al.* Methylammonium lead iodide perovskite-graphene hybrid channels in flexible broadband phototransistors. *Carbon N. Y.* **105**, 353–361 (2016).

11. Sun, Z., Aigouy, L. & Chen, Z. Plasmonic-enhanced perovskite-graphene hybrid photodetectors. *Nanoscale* **8**, 7377–7383 (2016).

12. Kwak, D.-H., Lim, D.-H., Ra, H.-S., Ramasamy, P. & Lee, J.-S. High performance hybrid graphene–$CsPbBr_{3-x}I_x$ perovskite nanocrystal photodetector. *RSC Adv.* **6**, 65252–65256 (2016).

13. Shao, Y. *et al.* Stable Graphene-Two-Dimensional Multiphase Perovskite Heterostructure Phototransistors with High Gain. *Nano Lett.* **17**, 7330–7338 (2017).

14. Betser, Y., Ritter, D., Liu, C. P., Seeds, A. J. & Madjar, A. A single-stage three-terminal heterojunction bipolar transistor optoelectronic mixer. *J. Light. Technol.* **16**, 605–609 (1998).

15. Gao, J. & Hegmann, F. A. Bulk photoconductive gain in pentacene thin films. *Appl. Phys. Lett.* **93**, 1–4 (2008).

16. Gong, X. *et al.* High-Detectivity Polymer Photodetector with Spectral Response from 300 nm to 1450 nm. *Science (80-.).* **325**, 1665–1667 (2009).

17. Li, F. *et al.* Ambipolar Solution-processed Hybrid Perovskite Phototransistors. *Arxiv Prepr.* **6**, 1–8 (2015).

18. Chen, C., Zhang, X., Wu, G., Li, H. & Chen, H. Visible-Light Ultrasensitive Solution-Prepared Layered Organic–Inorganic Hybrid Perovskite Field-Effect Transistor. *Adv. Opt. Mater.* **5**, 2–6 (2017).

19. Wu, X. *et al.* Trap states in lead iodide perovskites. *J. Am. Chem. Soc.* **137**, 2089–2096 (2015).

20. Leng, K. *et al.* Molecularly thin two-dimensional hybrid perovskites with tunable optoelectronic properties due to reversible surface relaxation. *Nat. Mater.* **17**, 908–914 (2018).

21. Feng, J. *et al.* Single-crystalline layered metal-halide perovskite nanowires for ultrasensitive photodetectors. *Nat. Electron.* **1**, 404–410 (2018).

22. Yu, W. *et al.* Single crystal hybrid perovskite field-effect transistors. *Nat. Commun.* **9**, 5354 (2018).

23. Kang, D. H. *et al.* An Ultrahigh-Performance Photodetector based on a Perovskite–Transition-Metal-Dichalcogenide Hybrid Structure. *Adv. Mater.* **28**, 7799–7806 (2016).

24. Park, Y. *et al.* Hybrid Metal-Halide Perovskite-MoS2 Phototransistor. *J. Nanosci. Nanotechnol.* **16**, 11722–11726 (2016).

25. Chen, Y., Chu, Y., Wu, X., Ou-Yang, W. & Huang, J. High-Performance Inorganic Perovskite Quantum Dot–Organic Semiconductor Hybrid Phototransistors. *Adv. Mater.* **29**, 1–8 (2017).

26. Zou, C. *et al.* A Highly Sensitive UV–vis–NIR All-Inorganic Perovskite Quantum Dot Phototransistor Based on a Layered Heterojunction. *Adv. Opt. Mater.* **6**, 1–9 (2018).

27. Labram, J. G. *et al.* Temperature-Dependent Polarization in Field-Effect Transport and Photovoltaic Measurements of Methylammonium Lead Iodide. *J. Phys. Chem. Lett.* **6**, 3565–3571 (2015).

28. Senanayak, S. P. *et al.* Understanding charge transport in lead iodide perovskite thin-film field-effect transistors. *Sci. Adv.* **3**, 1–11 (2017).

29. Qian, L. *et al.* A Solution-Processed High-Performance Phototransistor based on a Perovskite Composite with Chemically Modified Graphenes(SI). *Adv. Mater.* **29**, (2017).

30. Xie, C. & Yan, F. Perovskite/poly(3-hexylthiophene)/graphene multiheterojunction phototransistors with ultrahigh gain in broadband wavelength region. *ACS Appl. Mater. Interfaces* **9**, 1569–1576 (2017).

31. Chang, P. H. *et al.* Ultrahigh responsivity and detectivity graphene-perovskite hybrid phototransistors by sequential vapor deposition. *Sci. Rep.* **7**, 1–10 (2017).

32. Kang, D. H. *et al.* An Ultrahigh-Performance Photodetector based on a Perovskite–Transition-Metal-Dichalcogenide Hybrid Structure(SI). *Adv. Mater.* **28**, 7799–7806 (2016).

33. Liu, F. *et al.* Enhancement of photodetection based on perovskite/MoS_2 hybrid thin film transistor. *J. Semicond.* **38**, 034002 (2017).

34. Huo, C., Liu, X., Wang, Z., Song, X. & Zeng, H. High-Performance Low-Voltage-Driven Phototransistors through $CsPbBr_3$ -2D Crystal van der Waals Heterojunctions. *Adv. Opt. Mater.* **1800152**, 1800152 (2018).

35. Bao, Q. & Loh, K. P. Graphene photonics, plasmonics, and broadband optoelectronic

devices. *ACS Nano* **6**, 3677–3694 (2012).

36. Novoselov, K. S. *et al.* A roadmap for graphene. *Nature* **490**, 192–200 (2012).

37. Xie, C., You, P., Liu, Z., Li, L. & Yan, F. Ultrasensitive broadband phototransistors based on perovskite/organic-semiconductor vertical heterojunctions. *Light Sci. Appl.* **6**, e17023 (2017).

38. Johnson, N. M. & Chiang, A. Highly photosensitive transistors in single-crystal silicon thin films on fused silica. *Appl. Phys. Lett.* **45**, 1102–1104 (1984).

39. Spina, M. *et al.* Ultrasensitive 1D field-effect phototransistors: CH3NH3PbI3nanowire sensitized individual carbon nanotubes. *Nanoscale* **8**, 4888–4893 (2016).

40. Li, F. *et al.* Ultrahigh Carrier Mobility Achieved in Photoresponsive Hybrid Perovskite Films via Coupling with Single-Walled Carbon Nanotubes. *Adv. Mater.* **29**, 1602432 (2017).

41. Schwierz, F. Graphene transistors. *Nat. Nanotechnol.* **5**, 487–496 (2010).

42. Meric, I. *et al.* Current saturation in zero-bandgap, top-gated graphene field-effect transistors. *Nat. Nanotechnol.* **3**, 654–659 (2008).

43. Yu, Y. *et al.* Broadband Phototransistor Based on $CH_3NH_3PbI_3$ Perovskite and PbSe Quantum Dot Heterojunction. *J. Phys. Chem. Lett.* **8**, 445–451 (2017).

44. Yu, Y. *et al.* High Performances for Solution-Pocessed 0D–0D Heterojunction Phototransistors. *Adv. Opt. Mater.* **5**, 1–6 (2017).

45. Liu, X. *et al.* A highly sensitive and fast graphene nanoribbon/CsPbBr3quantum dot phototransistor with enhanced vertical metal oxide heterostructures. *Nanoscale* **10**, 10182–10189 (2018).

46. Schießl, S. P. *et al.* Hybrid Modulation-Doping of Solution-Processed Ultrathin Layers of ZnO Using Molecular Dopants. *Adv. Mater.* n/a-n/a (2015). doi:10.1002/adma.201503200

47. Kufer, D. & Konstantatos, G. Photo-FETs: Phototransistors Enabled by 2D and 0D Nanomaterials. *ACS Photonics* **3**, 2197–2210 (2016).

48. Buscema, M. *et al.* Photocurrent generation with two-dimensional van der Waals semiconductors. *Chem. Soc. Rev.* **44**, 3691–3718 (2015).

49. Chen, J. & Park, N.-G. Causes and Solutions of Recombination in Perovskite Solar Cells. *Adv. Mater.* **0**, 1803019 (2018).

50. Ran, C., Xu, J., Gao, W., Huang, C. & Dou, S. Defects in metal triiodide perovskite materials towards high-performance solar cells: origin, impact, characterization, and engineering. *Chem. Soc. Rev.* **47**, 4581–4610 (2018).

51. Kroeze, J. E., Savenije, T. J., Vermeulen, M. J. W. & Warman, J. M. Contactless Determination of the Photoconductivity Action Spectrum, Exciton Diffusion Length, and Charge Separation Efficiency in Polythiophene-Sensitized TiO_2 Bilayers. *J. Phys. Chem. B* **107**, 7696–7705 (2003).

52. Savenije, T. J., Ferguson, A. J., Kopidakis, N. & Rumbles, G. Revealing the dynamics of charge carriers in polymer:fullerene blends using photoinduced time-resolved

microwave conductivity. *J. Phys. Chem. C* **117**, 24085–24103 (2013).

53. Labram, J. G. *et al.* Charge transport in a two-dimensional hybrid metal halide thiocyanate compound. *J. Mater. Chem. C* **5**, 5930–5938 (2017).

54. Reid, O. G. *et al.* Quantitative analysis of time-resolved microwave conductivity data. *J. Phys. D. Appl. Phys.* **50**, 493002 (2017).

55. Levine, I. *et al.* Can we use time-resolved measurements to get steady-state transport data for halide perovskites? *J. Appl. Phys.* **124**, 103103 (2018).

56. Labram, J. G. & Chabinyc, M. L. Recombination at high carrier density in methylammonium lead iodide studied using time-resolved microwave conductivity. *J. Appl. Phys.* **122**, 065501 (2017).

57. Fang, Y., Armin, A., Meredith, P. & Huang, J. Accurate characterization of next-generation thin-film photodetectors. *Nat. Photonics* **13**, 1–4 (2019).

58. Liu, C.-H., Chang, Y.-C., Norris, T. B. & Zhong, Z. Graphene photodetectors with ultra-broadband and high responsivity at room temperature. *Nat. Nanotechnol.* **9**, 273–8 (2014).

59. Adinolfi, V. & Sargent, E. H. Photovoltage field-effect transistors. *Nature* **542**, 324–327 (2017).

60. Zhang, W. *et al.* Role of Metal Contacts in High- Performance Phototransistors Based. *ACS Nano* **8**, 8653–8661 (2014).

61. Shen, L. *et al.* A Self-Powered, Sub-nanosecond-Response Solution-Processed Hybrid Perovskite Photodetector for Time-Resolved Photoluminescence-Lifetime Detection. *Adv. Mater.* **28**, 10794–10800 (2016).

62. Zhu, H. L. *et al.* Room-Temperature Solution-Processed NiOx:PbI2Nanocomposite Structures for Realizing High-Performance Perovskite Photodetectors. *ACS Nano* **10**, 6808–6815 (2016).

63. Jeon, S. *et al.* Gated three-terminal device architecture to eliminate persistent photoconductivity in oxide semiconductor photosensor arrays. *Nat. Mater.* **11**, 301–305 (2012).

64. Liao, L. *et al.* High-speed graphene transistors with a self-aligned nanowire gate. *Nature* **467**, 305–308 (2010).

65. Zhang, H. *et al.* Short Channel Quantum Dot Vertical and Lateral Phototransistors. *Adv. Opt. Mater.* **5**, 1600434 (2017).

66. Adel S. Sedra & Kenneth C. Smith. *Microelectronic Circuits. Oxford University Press* (2007).

67. Traversi, F., Russo, V. & Sordan, R. Integrated complementary graphene inverter. *Appl. Phys. Lett.* **94**, 1–4 (2009).

68. Xiao, Z. *et al.* Giant switchable photovoltaic effect in organometal trihalide perovskite devices. *Nat. Mater.* **14**, 193-198 (2015).

69. Yuan, Y. *et al.* Anomalous photovoltaic effect in organic-inorganic hybrid perovskite solar cells. *Sci Adv* **3**, e1602164 (2017).

70. Hwang, B. & Lee, J. S. Recent Advances in Memory Devices with Hybrid Materials. *Adv. Electron. Mater.* **5**, 1800519 (2019).

71. Yuan, Y. *et al.* Ultra-high mobility transparent organic thin film transistors grown by an off-centre spin-coating method. *Nat. Commun.* **5**, 3005 (2014).
